# Spatiotemporal characteristics and driving forces of terrorist attacks in Belt and Road regions

**DOI:** 10.1371/journal.pone.0248063

**Published:** 2021-03-11

**Authors:** Lin Chen, Fengyun Mu

**Affiliations:** College of Architecture and Urban Planning, Chongqing Jiaotong University, Chongqing, China; Institute for Advanced Sustainability Studies, GERMANY

## Abstract

To achieve the strategic goals of the Belt and Road Initiative (BRI), it is necessary to deepen our understanding of terrorist attacks in BRI countries. First, we selected data for terrorist attacks in BRI regions from 1998 to 2017 from the Global Terrorism Database and analyzed their time distribution using trend analysis and wavelet analysis. Then, we used honeycomb hexagons to present the spatial distribution characteristics. Finally, based on the Fragile States Index, we used GeoDetector to analyze the driving forces of the terrorist attacks. The following conclusions were obtained: (1) During 1998–2017, the number of events was the highest on Mondays and the lowest on Fridays. In addition, the incidence of events was high between Monday and Thursday but was the lowest on Fridays and Saturdays. The number of events was the largest in January, May, July, and November and was the lowest in June and September; the incidence of terrorist attacks from April to May and July to August was high. (2) Terrorist attacks showed a 10-year cycle during the study period. Terrorist attacks in the last 10 years of the study period were broader in scope and higher in number compared with the previous 10 years. In addition, China, Russia, Saudi Arabia, and northeastern Europe saw many new terrorist attacks during the latter 10 years. (3) The number of terrorist attacks by bombing/explosion was the largest, followed by armed attack; assassination, kidnapping, and infrastructure attacks were the least frequent. The core areas of the terrorist attacks were Iraq, Israel, Afghanistan, Pakistan, and India. (4) The driving force analysis revealed that the indicators “security apparatus,” “human flight and brain drain,” and “external intervention” contributed the most to BRI terrorist attacks.

## Introduction

The Belt and Road Initiative (BRI) refers to both the “Silk Road Economic Belt” and the “21st Century Maritime Silk Road.” In 2013, Chinese president Xi Jinping proposed the construction of the “New Silk Road Economic Belt” as well as the “Cooperative Initiative of the Century Maritime Silk Road.” On the basis of existing regional cooperation platforms as well as bilateral and multilateral mechanisms between China and participating countries, the BRI (alluding to the ancient Silk Road) aims to achieve peaceful development and forge economic cooperation partnerships to build a community characterized by mutual trust, economic integration, and cultural tolerance. The BRI seeks to provide the international community with more public products through policy communication, facility connectivity, unimpeded trade, financial communication, and nongovernmental exchange. By sharing China’s development opportunities with BRI countries, mutual prosperity can be achieved, and sustainable development can be promoted [1]. That said, increases in terrorism events pose a serious obstacle to the smooth implementation of the BRI. Many serious terrorist attacks occurred in BRI countries between 1998 and 2017. For example, in the 2004 Beslan hostage incident in Russia, more than 1,100 hostages were taken, and 333 were killed. In 2008, a bomb attack in Mumbai, India, killed 195 people and injured 313. In 2014, a terrorist attack on the Kunming Railway Station in China killed 31 people and injured more than 141. In light of such events, investigating terrorist attacks in BRI countries can help deepen our understanding of terrorism and promote the realization of the strategic goals of the BRI.

Previous studies have examined the BRI from economic, environmental, and sustainability perspectives. Regarding economic research, Tao studied the effect of Chinese foreign direct investment (FDI) on exports in the BRI region and found an inverted U shape between Chinese FDI and host countries’ exports [2]. Meanwhile, You found that the levels of democracy in BRI regions exacerbated nonlinear relationships between income inequality and carbon dioxide emissions; moreover, various inequalities and poor democratic levels increased carbon dioxide emissions [3]. With regard to environmental studies, Hussain found that for every 1% increase in natural resource consumption in BRI countries, carbon dioxide emissions would increase by 0.0286%, and energy use would increase by 0.0117% [4]. Zhang found that from 2013 to 2015, the carbon emission intensity of BRI countries increased, with power-generation efficiency being the main influencing factor [5]. In terms of sustainable development, Huang suggested that BRI characteristics such as economic and environmental cooperation can promote cooperative sustainable development worldwide; however, resource utilization cooperation based on the fossil energy trade has adversely affected such global cooperation [6]. Although these previous studies analyzed the development status of BRI countries from multiple perspectives, security analyses are relatively rare. Terrorist attacks occur frequently in certain BRI countries, which can hinder the peaceful, cooperative development of the BRI. Therefore, adopting a security perspective, the present study investigated the internal relationship between terrorist attacks and the BRI.

It can be seen from the above materials that terrorist attacks will seriously interfere with national security and social stability, so the research on terrorist attacks can not be ignored and a number of studies have examined the impact of terrorist attacks. Dai, for example, analyzed the relationship between CEO salary increases and terrorist attacks, and found that CEOs employed at firms located near terrorist attacks earned an average pay increase of 12% after the attack relative to CEOs at firms located far from attacks [7]. Using the event research method, Bevilacqua studied terrorism’s effect on the volatility of U.S. financial markets. They found evidence of a greater impact of terror detected for the puts channel of volatility index (VIX), namely VIX−. They further showed that events that occurred within the U.S. appear to impact both VIX and VIX− in a similar way, whereas international terrorist attacks showed a greater impact on the puts component, VIX−. The calls component, VIX+, was found to be mainly detached from terrorist attacks [8]. Balcells assessed the effect of terrorism on public support for political parties and individuals’ willingness to participate in democratic elections and found that attacks were likely to increase individuals’ intent to participate in democratic elections but not to change their vote choice as reported in the surveys [9]. Meanwhile, Hussain, using the analytic hierarchy process, developed a quantitative method to measure the image of a country in reports on terrorism, it would help the future researchers to remove the biases while measuring significant country image quantitatively, which they faced in qualitative research [10]. Other studies have examined terrorism in terms of risk assessment and prediction. Khan constructed a model to measure the economic impact of terrorism on a country or region during a specific period. This model helped the policy makers to gauge the intensity of terrorism to measure at state level and used dynamic approach to examine economic cost at state level of any economy [11]. Zhang improved the traditional location recommendation algorithm by combining multisource factors and spatial characteristics. Considering 17 influencing factors, including socioeconomic and natural-resource factors, Zhang’s study established a spatial risk-assessment model for terrorist attacks, and found that the southernmost part of the Indochina peninsula and the Philippines are high-risk areas and that the medium-risk and high-risk areas are mainly distributed in the coastal areas [12].

Predicting the occurrence of terrorist attacks can detect hot spots of terrorist attacks within the scope of research in advance, and provide decision-making support for international counter-terrorism organizations and related countries to study the law of terrorist behavior and key prevention and control work. So the ability to predict terrorist attacks is important for prevention and response strategies. Hao used geospatial statistics to analyze the spatiotemporal evolution of terrorist attacks on the Indochinese peninsula. The results indicated that Thailand was the most dangerous area for terrorist attacks, especially southern Thailand, Bangkok and its surrounding cities. Middle Cambodia and the northern and southern parts of Myanmar were also high-risk areas [13]. To analyze and predict the spatial pattern of terrorist attacks, Buscema used spatial grammar to make complex inferences related to the deep structural layers of the data-generation process and its spatially distributed structure. And he tested the methodology on two of the currently most important and virulent theaters of terrorist activity, Libya and Syria, and found that the methodology provided a rich array of insights which cannot be expected to be generated by more traditional geographical profiling methods [14]. Li proposed a probabilistic analysis and prediction model for the conditions of terrorist attacks based on time series and used intervention analysis to examine sudden increases in the time series. The model was used to forecast the monthly conditional probability of bombing attacks in 2014 and through 2064. The average relative error compared with the real data in 2014 is 3.5%. It was also applied to the total number of attacks recorded by the GTD between 2004 and 2014 [15]. Based on 2006–2016 data from the Vulnerable Countries Index and Global Terrorism Database (GTD), Qiu used various machine-learning models to regress and predict the risk of terrorist attacks in various countries. The results showed that random forest, k-nearest neighbor and decision tree model performed best. However, the results of random forest prediction were generally in line with the actual situation, especially in the Middle East and Central Asia where terrorist attacks occur frequently [16].

Other studies have examined terrorism in terms of geographic distribution, internal time series connections, and related influencing factors. Based on 25 observation variables from the Global Terrorism Database, Hu used factor analysis to construct 11 factors related to the impact of terrorist attacks and identified 10 terrorist attacks with the most impact for a 20-year period [17]. Braithwaite used spatial statistics to identify terrorist hotspots and countries and evaluated the impact of these hotspots on the pattern of future terrorist incidents [18].

Regarding the driving force analysis method, GeoDetector has been widely used. It is a new tool to measure, mine, and utilize spatial heterogeneity [19]. The core of its theory is to detect the consistency of the spatial distribution pattern between the dependent variable and the independent variable through spatial heterogeneity and to measure the degree of interpretation of the dependent variable according to the independent variable (i.e., the q value). GeoDetector offers more advantages than general statistics because it is more difficult for two variables to be consistent in two-dimensional spatial distribution than in a one-dimensional curve of two variables. GeoDetector has been used in a wide range of fields from natural environment to social development phenomenon, including environmental sciences [20–22], soil science [23–25], groundwater nitrate contamination [26, 27], landslide [28, 29], PM2.5 [30, 31], remote sensing [32], land use [33–35], urban expansion [36], and public health [37–40]. Its research area can be large to the national scale [41–43] and small to a township scale [44, 45]. In these applications, geographic detectors are used mainly to analyze the driving force and influence the factors of various phenomena as well as the interaction of multiple factors. Therefore, its use also is appropriate for the analysis of the driving forces of terrorist attacks.

Although prior studies have examined terrorism from multiple perspectives, few have considered the spatiotemporal characteristics of terrorist attacks in detail. Most studies have either considered spatiotemporal characteristics in a limited way or simply have analyzed such characteristics without connecting them. In addition, because of the difficulty of collecting data from various countries around the world, there are relatively few studies of multisource factors affecting terrorist attacks. Therefore, adopting a spatiotemporal perspective, this study aimed to identify the distribution law of terrorist attacks in BRI regions while using the Fragile States Index for a driving-force analysis. From the perspective of the overall year, month-year, and week-year, we used trend analysis to analyze the number and annual incidence of terrorist attacks and used wavelet analysis to determine the distribution period. We also divided terrorist attacks into different periods. For visualization, we used ArcGIS to render statistics on the number of terrorist attacks in different periods into hexagonal unit cells. Based on the classifications of different attacks, we visualized the spatial distribution of attacks during the main time periods. Last, based on the Fragile States Index, we used GeoDetector to explore the drivers of terrorist attacks.

## Materials and method

### Data sources

We used the Global Terrorism Database (GTD; https://www.start.umd.edu/gtd/) to obtain terrorist attacks during the period 1998–2017. We derived the driving force index data for security, politics, economy, and society from the Fragile States Index (https://fragilestatesindex.org/). The objects of this study were BRI member states, which by 2017 included 65 countries.

#### Global terrorism database

Developed in the United States, the GTD records terrorist attacks reported in global media between 1970 and 2017 and is considered authoritative. For each terrorism event in the GTD, it is possible to query the date, location, weapons, targets, casualties, economic losses, and the responsible person or group, along with other information. For this analysis, we selected terrorist attacks occurring in BRI countries from 1998 to 2017.

#### Fragile States Index

The Fragile States Index is powered by the Fund for Peace, which works to prevent conflict and promote sustainable security worldwide by building relationships and trust among diverse sectors. Combining social science techniques with information technology, the Fund for Peace produced the patented Conflict Assessment System Tool, a content analysis application that provides a conceptual framework and data-gathering technique for measuring conflict risk. Annually, it produces the Fragile States Index, a ranking of 178 countries based on 12 indicators of risk and vulnerability. Table 1 presents the indicators.

**Table 1:** Fragile States Index.

Primary indicators	Secondary indicators	Abbreviations	Indicator descriptions
Cohesion indicators	Security apparatus	SA	Considers the security threats to a state, such as bombings, attacks and battle-related deaths, rebel movements, mutinies, coups, or terrorism.
	Factionalized elites	FE	Considers the fragmentation of state institutions along ethnic, class, clan, racial, or religious lines, as well as brinksmanship and gridlock between ruling elites.
	Group grievance	GG	Focuses on divisions and schisms between different groups in society—particularly divisions based on social or political characteristics—and their role in access to services or resources as well as inclusion in the political process.
Economic indicators	Economic decline	EC	Considers factors related to economic decline in a country.
	Uneven economic development	UD	Considers inequality within the economy, regardless of the actual performance of the economy.
	Human flight and brain drain	HF	Considers the economic impact of human displacement (for economic or political reasons) and the consequences it may have for a country’s development.
Political indicators	State legitimacy	SL	Considers the representativeness and openness of a government and its relationship with its citizenry.
	Public services	PS	Refers to the presence of basic state functions that serve the public.
	Human rights and rule of law	HR	Considers the relationship between the state and its population insofar as human rights are protected and freedoms are observed and respected.
Social indicators	Demographic pressures	DP	Considers pressures on the state deriving from the population itself or the environment around it.
	Refugees and internally displaced persons (IDPs)	RD	Measures the pressure on states caused by the forced displacement of large communities as a result of social, political, environmental, or other causes; it measures displacement within countries as well as refugee flows into others.
	External intervention	EX	Considers the influence and impact of external actors in the functioning—particularly security and economic—of a state.

### Methods

#### Wavelet analysis

The wavelet function can reveal change periods hidden in various time series and fully reflects the change trend in a time series at different time scales [46]. For a given Morlet wavelet and time series, the formula of CWT is

$${\text{W}}_{f}(\text{a},\text{b})=\frac{1}{\sqrt{a}}\int_{-\infty }^{+\infty }f(\text{t})\bar{\varphi }(\frac{t-b}{a})dt$$ (1)

where *W*_*f*_(*a*, *b*) is the wavelet transform coefficient, *φ*(*t*) is the Morlet mother wavelet, and $\bar{\varphi }$ is the complex conjugate of *φ*; *a* is the time scale, reflecting the periodic scale of the wavelet; and *b* is the translation factor, reflecting the translation in time.

#### Honeycomb hexagon

The traditional statistical unit is the square fishing net mesh. This study, however, used the honeycomb hexagon [47]. The honeycomb models can serve as a useful alternative to the classical square models for a broad class of problems. In some regards, they are “more economic” than those based on square models [48]. The honeycomb model has the following advantages:

1. It is the best topological structure covering a two-dimensional plane.
2. When the study area is large, compared with a fishing net grid, a hexagon grid reduces distortion caused by the curvature of the earth.
3. When using distance range to find a neighborhood, using optimized hotspot analysis, using optimized outlier analysis, or creating a spatiotemporal cube tool through aggregation points, the honeycomb hexagon is best since the distance from the hexagon to the center of mass is the same in all six directions.

#### GeoDetector

According to the theory that the spatial distribution of two variables tends to be consistent, Wang et al. added the spatial differentiation of the variables to detect the degree of influence on the spatial pattern distribution of the dependent variable. On this basis, the geographic detector model (GeoDetector) was developed [49]. This model is widely used in many research areas, such as land use, regional economies, and ecological environments. GeoDetector is mainly used to analyze the driving force of various phenomena. The q value of GeoDetector has a clear physical meaning. It can objectively detect 100*p% dependent variables, which are explained by independent variables. GeoDetector is not based on linear assumptions, but rather it compares the spatial consistency of independent variable distribution with the geographical strata in which the potential factors exist [19].

(1) Factor detector.
  In the present study, we used GeoDetector to detect the explanatory power of the Fragile States Index for the number of terrorist attacks. The model formula is as follows:
  $$q=1-\frac{\sum_{i=1}^{L}N_{i}\sigma_{\text{i}}^{2}}{{\text{N}\sigma }^{2}}$$ (2)
  where *i* = 1, …, *L* is the classification number of indicators, *Ni* is the number of class *I* samples, *n* is the number of samples in the study area, *σ*^*2*^_*i*_ is the variance of class *I*, and *σ*^*2*^ is the variance in the number of terrorist attacks. The value range of explanatory power *q* is [0,1]. The larger the value of *q*, the stronger the explanatory power of the index for the number of terrorist attacks, and vice versa.
(2) Interaction detector.
  Interaction detector assesses whether the explanatory powers of two factors are enhanced, weakened, or independent of each other. First, we calculated the *q* values of two factors X_1_ and X_2_ for Y (q(X_1_) and q(X_2_)). Then, we calculated the *q* value of the interaction (q (X_1_∩X_2_)), which was a new layer formed by the tangent of the overlay variables X_1_ and X_2_, and compared this value with q(X_1_) and q(X_2_) to indicate the interaction type between the two variables. The introduction of the five major types of interaction is shown in Table 2.

**Table 2:** Interaction between factors and their expressions.

Interaction type	Expression
Nonlinear-enhance	q(X1∩X2) #x003E; q(X1+q(X2)
Independent	q(X1∩X2) = q(X1+q(X2)
Bi-enhance	q(X1∩X2) > Max(q(X1), q(X2))
Uni-weaken	Min(q(X1), q(X2)) < q(X1∩X2) < Max(q(X1), q(X2))
Nonlinear-weaken	q(X1∩X2) < Min(q(X1), q(X2))

## Results

### Time characteristic analysis

**Fig 1:**
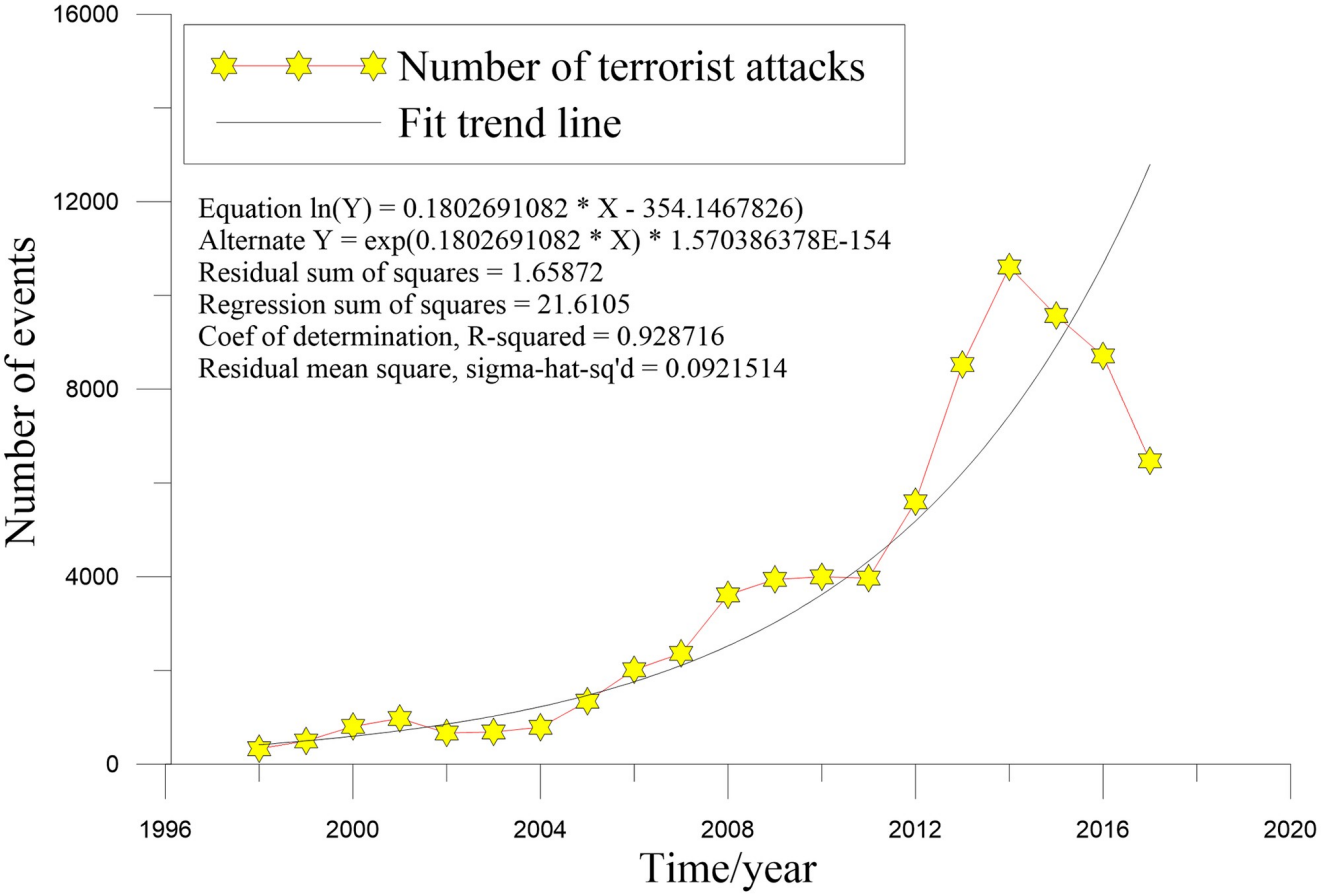
Number of terrorist attacks in BRI regions, 1998–2017. The black curve is a fitting line.

As depicted in Fig 1, the number of terrorist attacks in BRI countries showed an overall rising trend with a three-stage fluctuation growth trend. This result was consistent with Clauset’s view that the occurrence of terrorist attacks tends to accelerate with increased scale and experience [50]. In the first stage, from 1998 to 2004, the number of terrorism events fluctuated around 1000, showing the characteristics of first increasing and then decreasing. In 2001, there was a wave peak (i.e., 1682 events), with the September 11 terrorist attack in the United States being the representative event of that year. In the second stage, from 2004 to 2011, the number of events fluctuated even more, from 982 events in 2004 to 4344 events in 2008; the number of events then became flat over the next three years. The third stage was from 2011 to 2017, with an average annual increase of 2955 events from 2011 to 2014, reaching a maximum in 2014. Then, there was a turning point, with an average annual reduction rate of 1635 events, showing a rapid downward trend.

**Fig 2:**
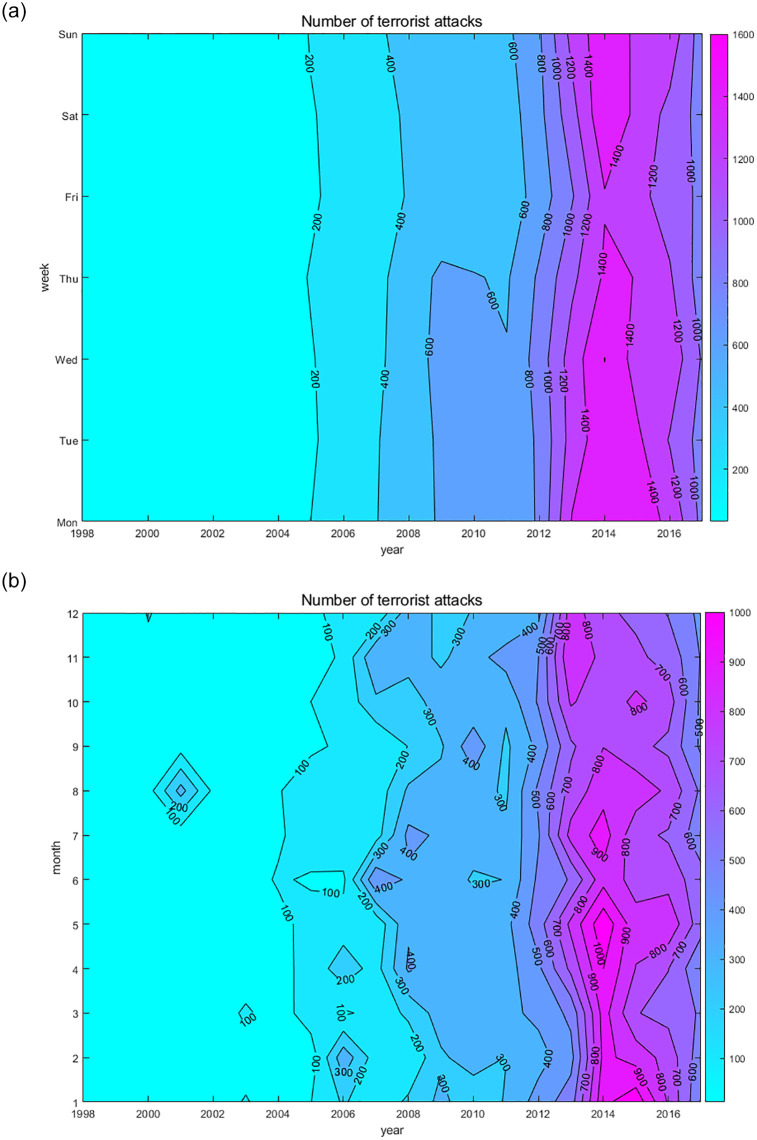
Contour map of the number of terrorist attacks. The darker the color, the higher the number of events. (a) shows the week-year relationship of the number of events. (b) shows the month-year relationship of the number of events.

**Fig 3:**
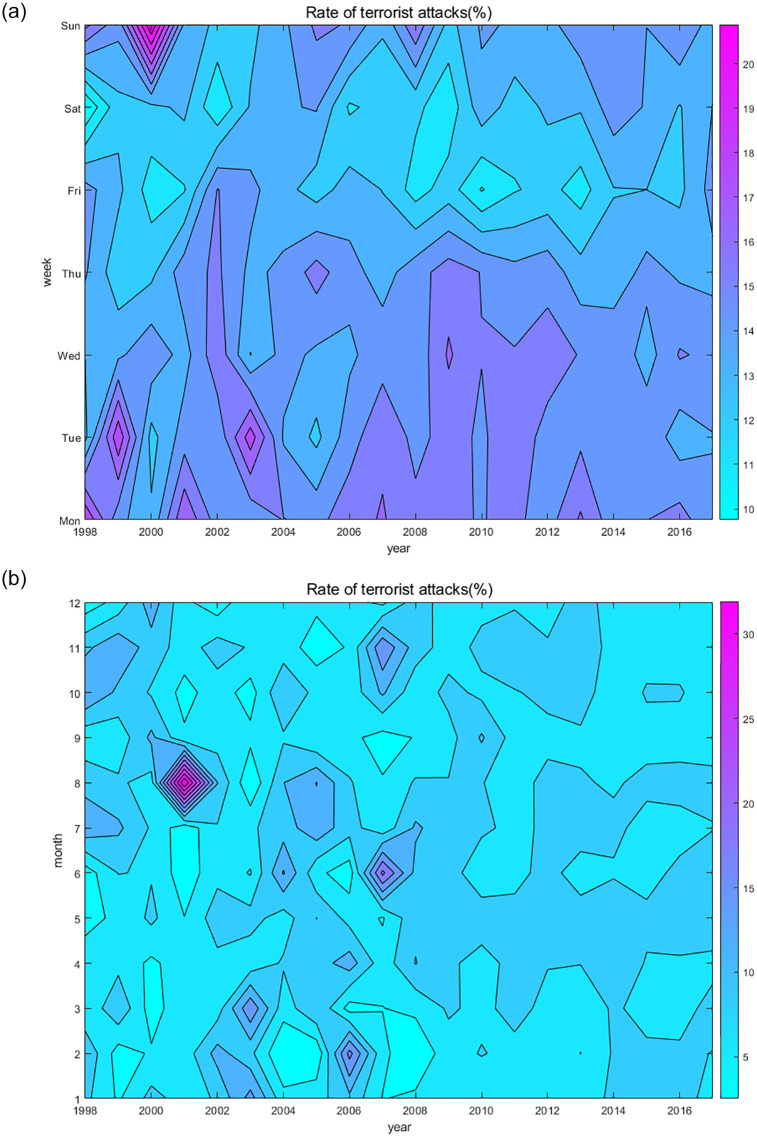
Contour map of the incidence of terrorist attacks. The darker the color, the higher the incidence of events in the year, (a) shows the week year relationship of the incidence of events. (b) shows the month year relationship of the incidence of events.

To reveal the overall distribution trend, we used Excel’s pivot table to count the number of BRI terrorist attacks occurring from Monday to Sunday (a year was divided into seven time periods; that is, “Monday” represents all Mondays in the year, “Tuesday” all Tuesdays, and so on) and January to December over the 20-year period. Then, to show the distribution characteristics of terrorist attacks every year, we calculated the annual incidence of terrorist attacks in BRI countries from Monday to Sunday and January to December. Finally, we used MATLAB to draw the week-year and month-year contour plots, as shown in Figs 2 and 3, respectively.

The characteristics of the number of terrorist attacks from Monday to Sunday and from January to December are similar to those of the total number, which can be divided into three stages. Fig 2(a) shows that during the first stage, 1998–2004, the number of terrorist attacks from Monday to Sunday was about 200, with a relatively uniform distribution. In the second stage, 2005–2014, the number of terrorist attacks increased with the increase of the year from Monday to Sunday, reaching a peak in 2014, among which the distribution of terrorist attacks in 2005–2008 and 2012–2013 from Monday to Sunday was relatively uniform. Monday to Thursday in 2009–2011 was the peak period for terrorist attacks, while Friday to Sunday was the low period. In addition, there were at least 1200 incidents for each period in 2014, representing the 20-year peak. The Friday period in 2014 (i.e., all Fridays that year), however, saw the lowest number of terrorist attacks, while the remaining periods saw more than 1400 each, with the Monday and Wednesday periods being the highest. In the third stage, 2015–2017, the number of terrorist attacks decreased with the increase of the year from Monday to Sunday. In 2015, the peak periods were Monday/Tuesday and Saturday/Sunday; by contrast, the low period was Friday. In 2016, the peak periods were Monday and Sunday, and Friday was the low period. In 2017, the distribution of the number of terrorist attacks was relatively uniform, and the peak period was Wednesday.

Fig 3(a) shows the weekly annual terrorist attack rate. Generally speaking, the figure can be divided into three parts, and the terrorist attack rates from Monday to Thursday and on Sunday during 1998–2017 were generally high, whereas those on Friday and Saturday were very low. Monday in 1998, Tuesday in 1999, Sunday in 2000, Monday in 2001, Tuesday in 2003, and Wednesday in 2009 had the highest incident rates (17–20%). The lowest incident rates, around 10–11%, were for Saturday in 1999, Tuesday and Friday in 2000, Saturday in 2001, Tuesday in 2005, Friday and Saturday in 2008–2009, Friday in 2010, and Friday in 2012.

Fig 3(b) shows the month-year occurrence rate of terrorist attacks. The monthly occurrence rate distribution from 1998 to 2008 was relatively random. The rate was less than 5% for May and June 1998; January and February 1999; March and April 2000; May, June, July, and October 2001; August and October 2003; February 2004; February and November 2005; March and June 2006; September 2007; and February 2008. Meanwhile, some months were over 15%, including 34% in August 2001, 20% in June 2007, and over 15% in January and March 2003, February 2006, and November 2007. The monthly incident rate for 2009–2017 was relatively average (about 5–10%) with the rate in April and May being relatively high (close to 10%). In addition, the incident rates for May and September 2009–2010, July and August 2012–2017, January and February 2014–2017, and October and November 2010–2013 were all close to 10%.

**Fig 4:**
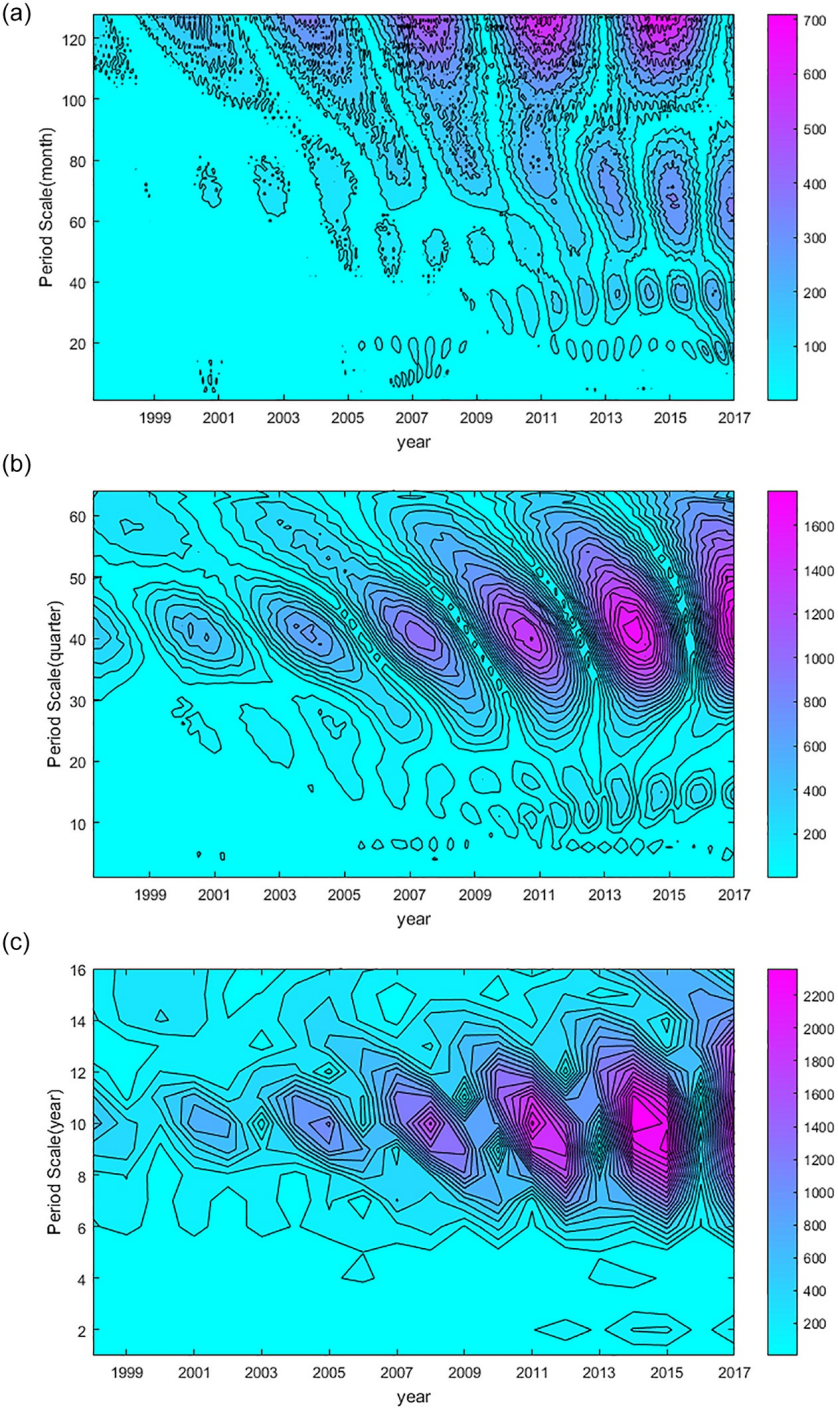
Graph of wavelet analysis results. (a)–(c) show the periodic oscillation characteristics of the change in the number of BRI terrorist attacks during the 20-year period at three scales: Month, quarter, and year. At time *x*, the darker the color, the more significant the characteristic of period *y*.

We used wavelet analysis to examine the periodic oscillation characteristics of the change in the number of BRI terrorist attacks during the 20-year period at three scales: month, quarter, and year. Fig 4 shows the results.

1. Fig 4(a) shows that the periods between 60 and 80 months in 2001–2017 and between 32 and 40 months in 2011–2017 were more significant. Moreover, the period between 120 and 125 months was the most significant in 1998–2017; the cycle characteristics become increasingly obvious over time.
2. In Fig 4(b), the cycle characteristics of 10–15 quarters in 2011–2017 were more obvious. Furthermore, the cycle characteristics of 40 quarters in 1998–2017 were the most significant, and the cycle characteristics increased over time.
3. In Fig 4(c), the 10-year-cycle characteristic of terrorist attacks in BRI countries from 1998 to 2017 was relatively significant and becomes increasingly obvious over time. In summary, our analysis of the cycle characteristics of terrorist attacks at three scales (month, quarter, year) showed that the 10-year-cycle characteristics of terrorism events in BRI countries from 1998 to 2017 were significant. Therefore, we divided the full 20-year period of terrorism events into two stages: 1998–2007 and 2008–2017.

### Spatial features analysis

**Fig 5:**
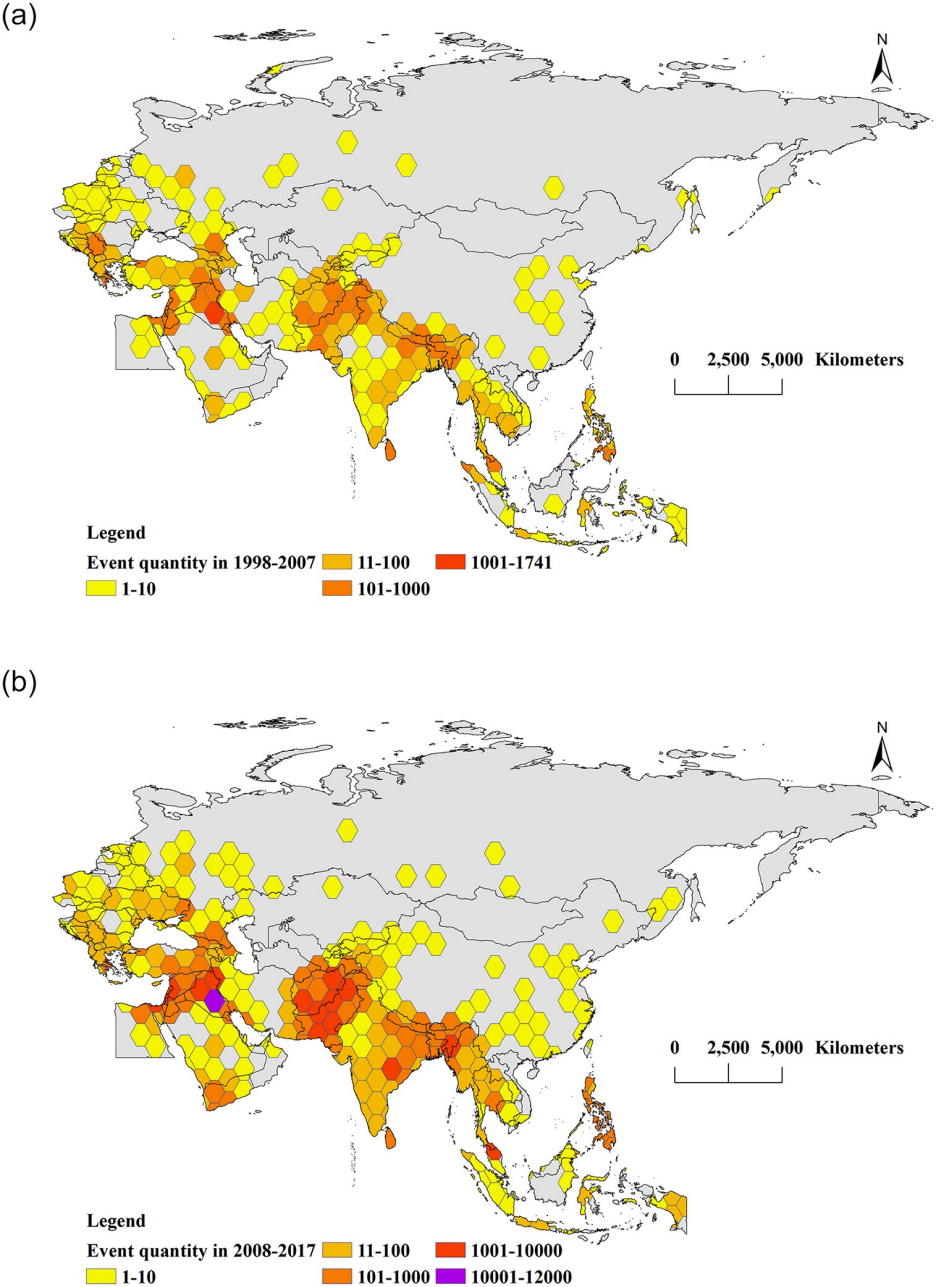
Spatial distribution of the number of terrorist attacks in BRI areas (made with Natural Earth). (a) shows the terrorist attacks during 1998–2007, and (b) shows the terrorist attacks during 2008–2017. Yellow honeycomb: 1–10; dark yellow honeycomb: 11–100; orange honeycomb: 101–1000; red honeycomb: 1001–10,000; violet honeycomb: >10,000.

From 1998 to 2017, there were 75,478 terrorist attacks in BRI countries. Of these, 10,467 occurred between 1998 and 2007, accounting for 13.9% of the total, and 65,011 occurred between 2008 and 2017, accounting for 86.1% of the total. We used ArcGIS to visualize these numbers on maps. Based on a cellular hexagon with an area of 120,000 km^2^ as the basic cell, we counted the number of terrorist attacks in each cell and obtained the spatial distribution of terrorism events for the two 10-year periods (Fig 5).

As shown in Fig 5(a), from 1998 to 2007, the more than 100 honeycomb hexagons of BRI terrorist attacks were mainly located in the Middle East, South Asia, Southeast Asia, and Southern Europe. In South Asia, this included the areas bordering Afghanistan and Pakistan, India-controlled Kashmir, northeastern India, eastern Nepal, and Sri Lanka. The regions in Southeast Asia, including southern Thailand, northern Indonesia, and southern Philippines, were all bordered by the sea. In the Middle East, including Israel, Palestine, and Lebanon on the east coast of the Mediterranean Sea, as well as northern and southeast Iraq, the red honeycomb hexagons, where 1,741 terrorism events occurred, were located in central Iraq. The honeycomb hexagons with more than 100 terrorist attacks in Europe were located in Serbia and southern Greece near the Mediterranean and in northern Georgia in southwestern Russia near the northern Caspian Sea.

Fig 5(b) shows that from 2008 to 2017, the honeycomb hexagons with more than 100 terrorist attacks in BRI countries were mainly concentrated in the Middle East, South Asia, Southeast Asia, and the eastern part of southern Europe. In the Middle East—including eastern Turkey, Syria, Iraq, Lebanon, Israel, Palestine, Jordan, Kuwait, Georgia, and southern Yemen near Somalia—there were more than 1000 terrorist attacks in Lebanon, Israel, and Palestine. The red honeycomb hexagon in northern Iraq and purple honeycomb hexagon in central Iraq indicated more than 10,000 terrorist attacks in Iraq. In South Asia—including Nepal, Bangladesh, eastern and northeastern India, western Myanmar, and Sri Lanka—red honeycomb hexagons appeared in eastern and northeastern India, indicating more than 1000 terrorist attacks. Note that both India and Bangladesh border the Indian Ocean. In Southeast Asia, a relatively large number of terrorism events occurred in southern Thailand, the Philippines, and central Malaysia, all of which were near the sea. Among them, more than 1000 terrorist attacks occurred in central Malaysia. Europe included northern Georgia in southwestern Russia near the west coast of the Caspian Sea as well as southern Greece. The honeycomb hexagons indicating more than 100 terrorist attacks in Egypt were located in the northeast region, close to the Mediterranean Sea. In Europe as a whole, areas with more than 100 incidents were declining. Meanwhile, areas with 10–100 incidents were increasing and mainly were concentrated in western Europe. Moreover, honeycomb hexagons indicating more than 100 events were located in northern Georgia and near the west coast of the Caspian Sea in southwestern Russia and in southern Greece.

In general, during 1998–2017, there were more terrorist attacks in the Middle East and South Asia, with the largest number of honeycomb hexagons located in Iraq. Compared with the first 10-year period, terrorist attacks in the latter period were wider in scope and larger in number. In the latter 10 years, most of the red honeycomb hexagons (more than 1000 events) expanded and spread on the basis of the orange honeycomb hexagons (more than 100 events) of the first 10 years. The same held true for the purple honeycomb hexagons (more than 10,000 events) in the latter 10 years, which expanded and spread on the basis of the red honeycomb hexagons of the previous 10 years. Note that most of the incident-prone areas were close to water, which to some extent suggested a connection between terrorism events and traffic.

**Fig 6:**
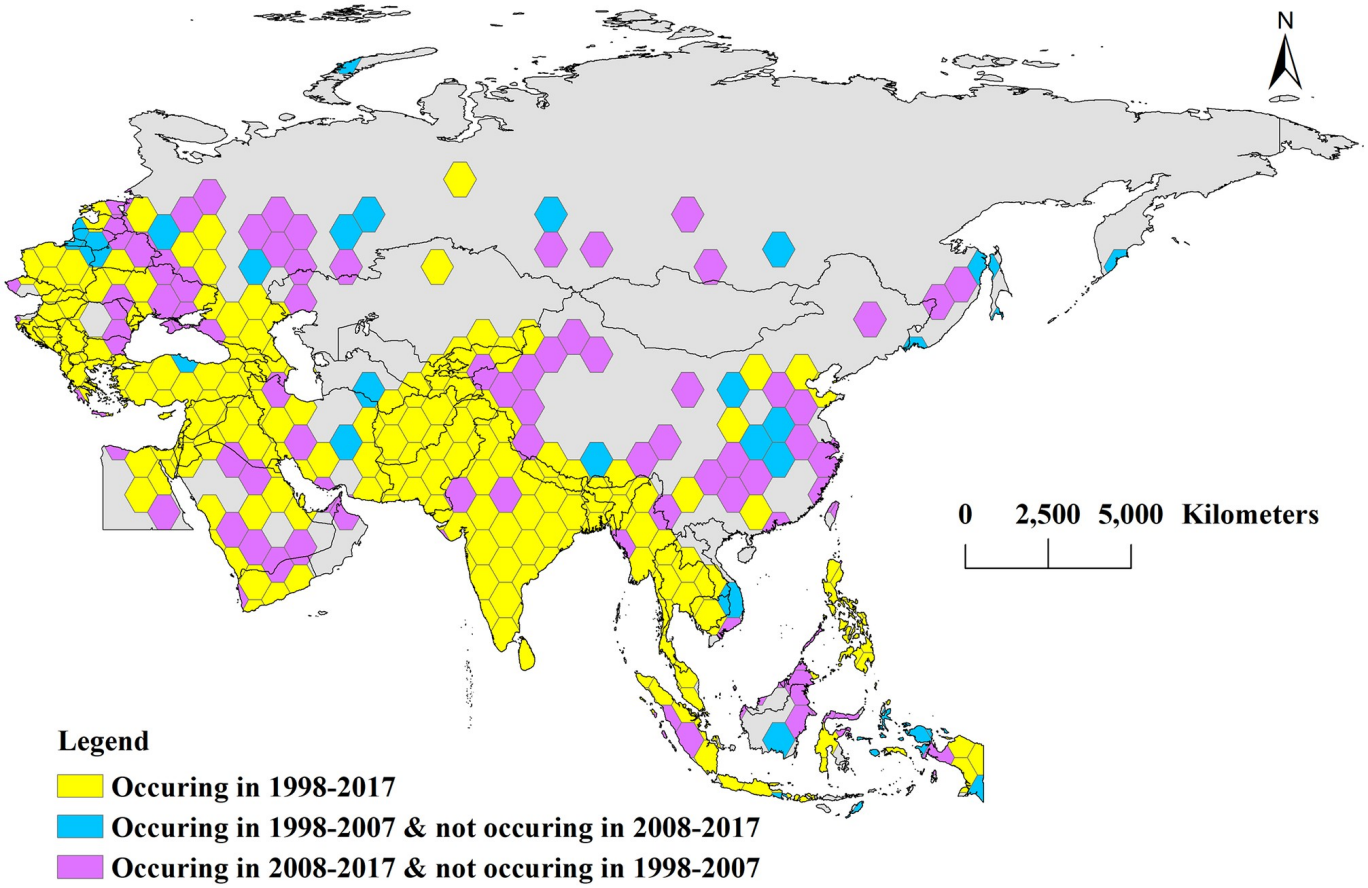
Time distribution of terrorist attacks in Belt and Road regions (made with Natural Earth). Yellow honeycomb: Occurring in 1998–2017; blue honeycomb: Only occurring in 1998–2007; violet honeycomb: Only occurring in 2008–2017.

We used cellular hexagons to show terrorist attacks that occurred during 1998–2007, 2008–2017, and 1998–2017, revealing the spatial distributions during the first 10 years, the latter 10 years, and the full 20-year period (Fig 6). Among BRI countries, terrorist attacks occurred frequently in the Middle East, South Asia, and Southeast Asia. These regions did not change much between the two 10-year periods, with most showing yellow honeycomb hexagons. Terrorism events in China, however, increased a great deal in the latter 10-year period. Nonetheless, the overall number of events in China during the full 20-year period was low. Meanwhile, terrorism events increased in Egypt during the latter period, expanding from the northeast during the first 10 years to the north and southeast during the next 10 years. In Europe, the yellow honeycomb hexagons are mainly concentrated in the west, with increases in the northeast and northern regions during the latter decade. By contrast, Moscow had more terrorist attacks in the latter 10-year period. Finally, Saudi Arabia had events in the southern, western, and northern regions during the first 10-year period, whereas the central region saw increased terrorist attacks during the second period.

### Spatiotemporal distribution characteristics of terrorist attacks by different categories

**Fig 7:**
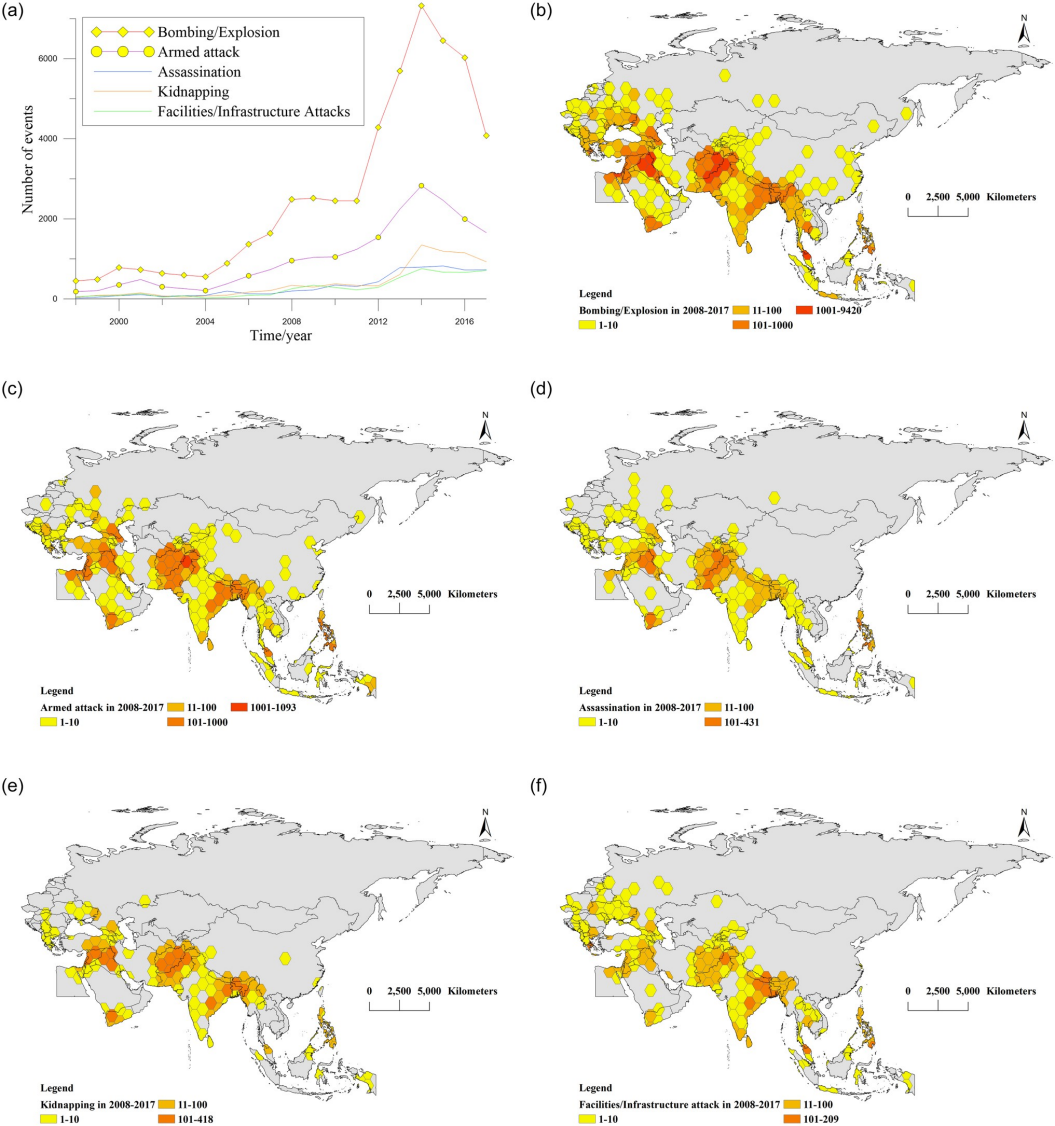
Spatial and temporal distribution of the five attack methods (made with Natural Earth). (a) shows the time curve of five attack methods. (b)–(f) show the spatial distribution of the five attack methods. Yellow honeycomb: 1–10; dark yellow honeycomb: 11–100; orange honeycomb: 101–1000; red honeycomb: 1001–10,000.

We divided terrorist attacks in BRI countries from 1998 to 2017 into five categories based on attack methods: bombing/explosion, armed attack, assassination, kidnapping, and facility/infrastructure attack. Their time curves were created, as shown in Fig 7(a). Because terrorism events in BRI countries follow a 10-year cycle, and the number in 2008–2017 accounted for 86.1% of the total, we used ArcGIS to visually express the spatial distribution of the five attacks in the latter 10 years, as shown in Fig 7(b)–7(f).

Fig 7(a) shows that bombing/explosive attacks were the largest in number. This method rose after 1998, dropped in 2000, increased again in 2004–2008, and then maintained a steady trend for the next four years. It then rose rapidly in 2011–2014 and declined rapidly afterward. Armed attack ranked second in the number of terrorism events. It increased first and then decreased from 1998 to 2004, peaking in 2001. Then, it gradually increased from 2004 to 2014, peaked in 2014, and rapidly declined afterward. Last, assassination, kidnapping, and facility/infrastructure attacks had the lowest numbers and showed the fewest changes. Among them, kidnapping slowly increased from 1998 to 2014 and then declined. Assassination and facilities/infrastructure attacks slowly accelerated from 1998 to 2013 and maintained a steady increasing trend from 2013 to 2017.

Fig 7(b)–7(f) show that among BRI countries, the five types of terrorist attacks were mainly distributed in the Middle East and South Asia. The core areas were Iraq/Israel, Afghanistan/Pakistan, and northeast India. The difference between them lay in the number of core areas and the size of the surrounding areas. Bombing/explosion events had the largest number of core areas and were distributed throughout India, most of the Arabian Peninsula, Southeast Asia, and countries neighboring northern Pakistan. Compared with bombings/explosions, the number and scope of armed attacks decreased. In addition to the core area, armed attacks were also distributed in eastern India and southern Saudi Arabia. The other three types of attacks had the lowest numbers, with few events seen in Yemen and southern India. In Egypt, the five types of terrorist attacks occurred mainly in the central region. Finally, in Europe, bombings/explosions and facility/infrastructure attacks were the most numerous, distributed in eastern and southern Europe. Armed attacks, assassinations, and kidnappings were the fewest, mainly concentrated in southern Europe.

### Driving force analysis

**Fig 8:**
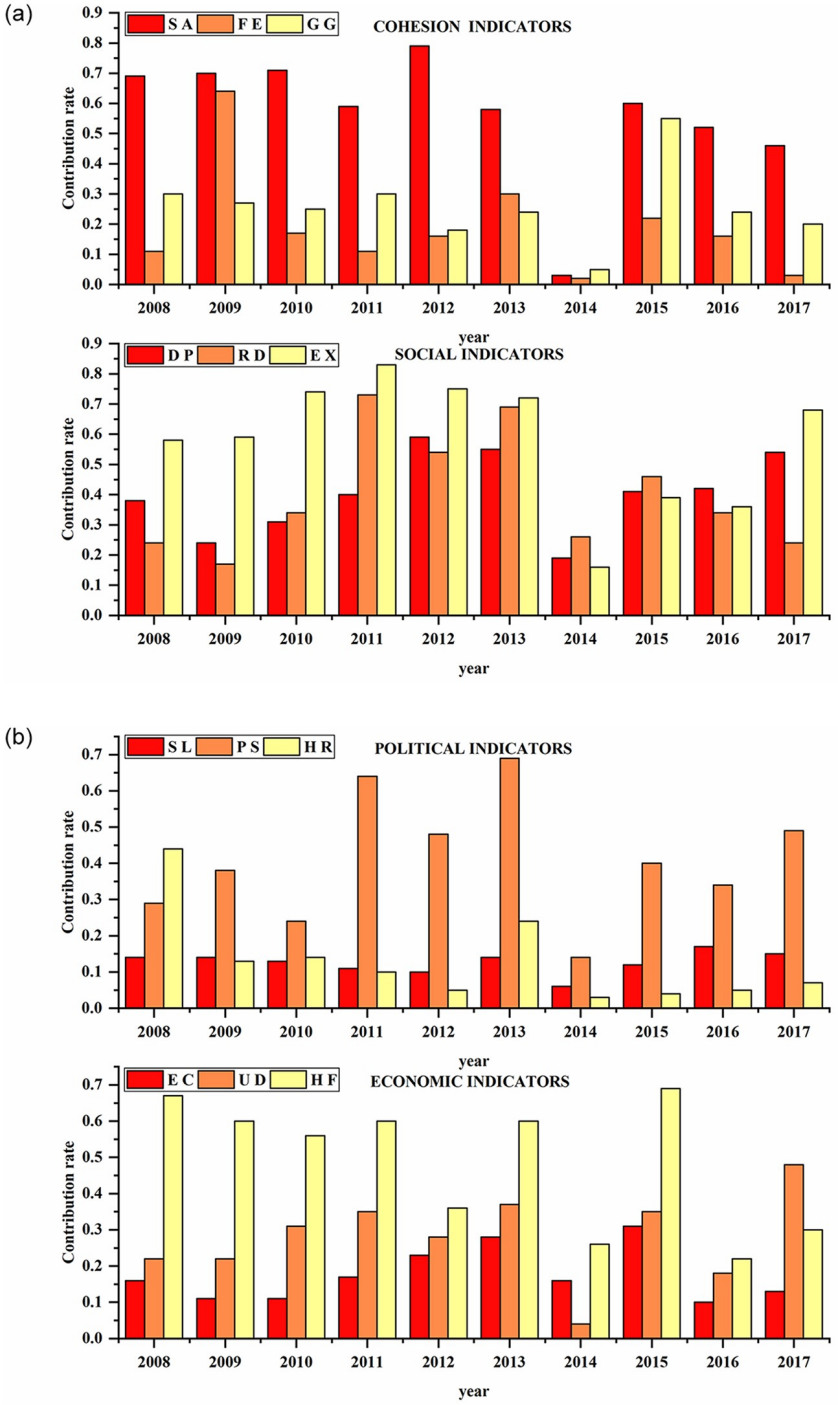
Driving force results. The contribution of the 12 indicators in the Fragile States Index to the number of terrorist events during 2008–2017.

We used the factor detection function in GeoDetector to calculate the contribution of the 12 indicators in the Fragile States Index to the number of terrorist events during 2008–2017. Fig 8 shows the results.

1. Among the security indicators, security apparatus’ contribution was the highest in all years, with a contribution rate of more than 0.6 in five years, reaching a maximum of 0.8. This could be because the indicator takes into account security threats to a country, such as explosions, attacks, battle-related deaths, rebellions, and coups, which tend to be directly related to terrorist attacks. The contribution of the factionalized elites was 0.65 in 2009, ranking second and remaining low for the rest of the period. By contrast, group grievance’s contribution ranked last in 2009 and ranked second for the rest of the period. This could be because the group grievance index focuses on differences and divisions between different groups in society, especially social or political differences, and how such divisions affect obtaining resources or participating in political processes. When certain groups, especially religious groups, or individuals feel wronged or oppressed, they are more prone to commit crimes, which can lead to terrorist incidents.
2. Among the political indicators, except for 2008, the contribution of public services was the largest in the remaining years, reaching 0.7 in 2013. This indicator includes two aspects. First, it can include the provision of basic services such as health care, education, water, sanitation, transportation infrastructure, electricity, and Internet. A country’s public service capacity is related to people’s happiness in daily life. When basic services are insufficient, the resulting pressures can give rise to crime or terrorism. Second, public services may include the state’s ability to protect citizens from terrorism and violence through effective police services, which is also directly related to the occurrence of terrorism. The contribution of other the two indicators—state legitimacy and human rights and rule of law—is low, indicating that they are not significantly related to terrorist attacks.
3. Among the social indicators, the contribution of external intervention in 2008–2013 and 2017 was the highest, ranging from 0.6 to 0.9. External intervention focuses on security aspects that may affect the balance of power among the actors involved in a country’s affairs. External intervention also considers humanitarian intervention. Today, with accelerated globalization processes, many terrorist attacks have occurred across national borders, deepening the connection between external intervention and terrorism. For example, the terrorist attack on a railway station in Xinjiang, China, was planned outside the country and carried out within the country. Moreover, the contribution of demographic pressures and refugees and IDPs was less than 0.5 most of the time. The contribution of population pressure was 0.5–0.6 in 2012, 2013, and 2017, whereas that of refugees and IDPs was 0.5–0.7 during 2011–2013. The population stress indicator takes into account the pressure on a country caused by the population or its surrounding environment. On the one hand, some countries have problems because of their own population characteristics, such as high growth rates, unbalanced distribution, and aging, which create political and economic pressure; this in turn affects people’s livelihoods, which can produce crime. On the other hand, some countries experience serious natural disasters, and the government may have inadequate response mechanisms; people’s lives are thus adversely affected, which may lead to crime or terrorism. The refugees and IDPs indicator measures refugees by country of asylum, recognizing that population inflows can put additional pressure on public services. This can sometimes create humanitarian and security challenges for the receiving state if it lacks absorption capacity and adequate resources. This indicator also measures refugees and IDPs by country of origin, which signifies internal state pressures as a result of violence, environmental factors, or other factors, such as epidemics. Therefore, a larger value for this index could affect the occurrence of terrorist attacks. In general, the three social indicators had relatively large contributions in 2011–2013. Similarly, the number of terrorist attacks increased at a maximum rate in 2011–2014, which once again showed that these three indicators had a greater correlation with the occurrence of terrorist attacks. It is likely that these three indicators will have a certain lag influence, resulting in the number of terrorist attacks in 2014 continuing to rise to the highest level over the years.
4. Among economic indicators, human flight and brain drain had the highest contribution for six years, between 0.55 and 0.7. On the one hand, brain drain can involve the voluntary immigration of the middle class. On the other hand, it can involve the forced displacement of professionals or intellectuals who flee a country because of persecution or repression. When a country’s economic development capacity is low, and the loss of national talent leads to underdevelopment, people may find it difficult to survive, which can lead to crime and even terrorism. The contribution of economic decline was low. It focused on decline in terms of per capita income and gross national product. If a country’s economic level was already low, the room for decline was small, but this did not express people’s quality of life. The contribution of uneven economic development ranked second. The gap between rich and poor, along with unfair economic systems, can cause oppressed or disadvantaged people to turn to crime or terrorism.

Overall, the top three indicators were security apparatus, human flight and brain drain, and external intervention. Most indicators, however, were abnormally low in 2014. This could be because the number of terrorist attacks was the highest ever, and other potential factors affected it.

### Interaction analysis

**Fig 9:**
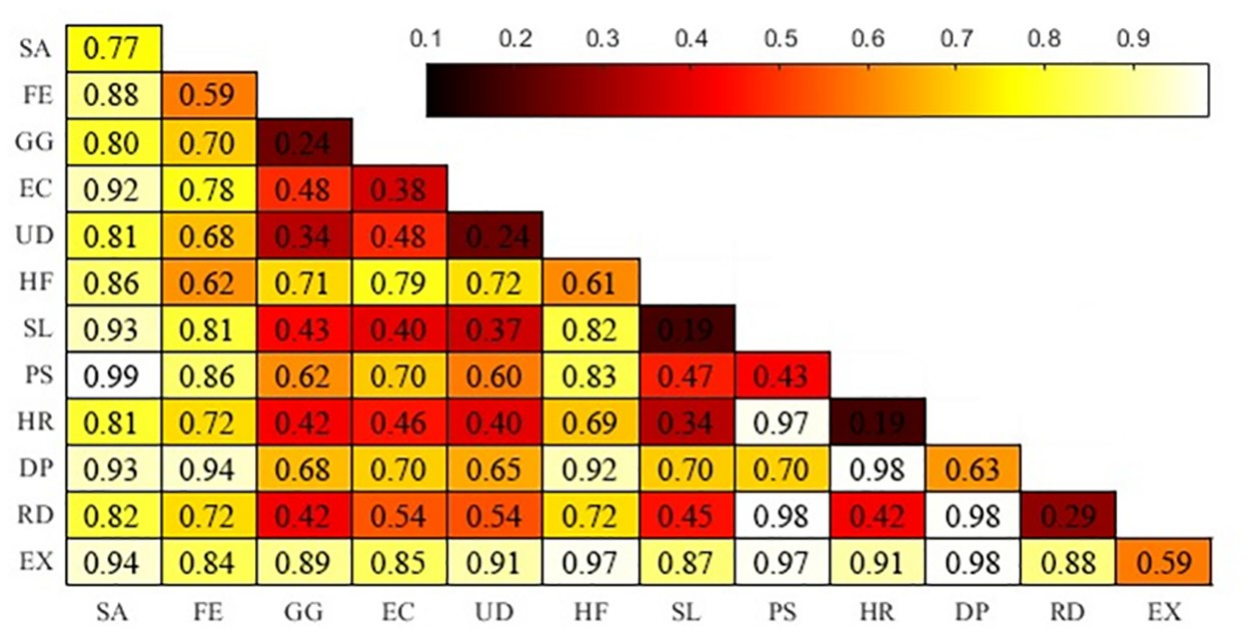
The value of interaction between factors. The brighter the color, the higher the value.

From the interaction detection of Fragile States Index, we obtained the common explanatory power of every two factors for terrorist attacks (the results are shown in Fig 9). The interaction of any two factors was double enhancement or nonlinear enhancement. In these interactions, the interaction between Security apparatus, External intervention, and all indicators was higher than 0.8, which once again confirmed that Security apparatus and External intervention were the strong influencing factors of BRI terrorist attacks. In addition to these two factors, the interaction between Public services, Refugees and IDPs, and Human rights was greater than 0.97. Relevant research has shown that the equalization of health care and education significantly reduces the criminal crime rate [51]. Moreover, a harmonized definition of terrorist offenses is very important. The European Union has formulated many criminal laws on terrorist attacks [52]. The interaction between Refugees and IDPs and Demographic pressures was 0.98. This reflected the following: if the population pressure of a country was too large, or if basic social welfare could not be guaranteed, it inevitably would lead to an increase in refugees, and then lead to an increase in the number of criminals.

## Discussion

### Two 10-year discoveries

Wavelet analysis has been used widely to discover the distribution characteristics of time series and to find its time resolution [53, 54]. Therefore, we used wavelet analysis to examine the periodic oscillation characteristics of the change in the number of BRI terrorist attacks during the 20-year period at three scales: month, quarter, and year. Our analysis of the cycle characteristics of terrorist attacks at three scales (month, quarter, year) showed that the 10-year-cycle characteristics of terrorism events in BRI countries from 1998 to 2017 were significant. We found an interesting pattern that BRI terrorist attacks have occurred mainly in the past 10 years. In addition, the Fragile States Index was available only for 2005–2019. Thus, we decided to shift our research focus to BRI terrorist attacks in the past 10 years.

### Distribution characteristics in space and time

Terrorist attacks mainly occurred on weekdays, but less on weekends, which was the opposite of aggressive crime. Weekends generally had higher mean aggressive crime counts than weekdays, supporting the idea that interactions between people, likely outside of the workplace, would lead to the opportunity for higher amounts of aggressive crime to occur [55]. To disrupt the normal social order and achieve some special purpose, such as an assassination and panic, terrorist attacks were more frequent on weekdays. In addition, there were seasonal differences in crime. A variety of criminal incidents occurred from June to October. Similarly, we found that the incidence of terrorist attacks was higher in summer and autumn, especially in the past 10 years. This phenomenon might be related to temperature to a certain extent, that is, the higher the temperature, the more suitable for crime [56].

Notably, criminal activities were closely related to the surrounding environment, and these activities were more likely to occur in places with convenient transportation [57]. We found that terrorist attacks were prone to occur in coastal countries, which might have been due to the fact that some terrorist attacks were carried out transnationally, so better traffic conditions were more suitable.

### Driving forces

We found the top driving force indicators were security apparatus and external intervention. In Southeast Asia, widespread corruption created conditions for secret transactions between terrorists and government officials, so the government was more likely to be interrupted by external forces. In addition, organized criminal activities, such as human smuggling and passport counterfeiting, which were active in Southeast Asia, created cross-border movements of terrorists, which seriously threatened national security [58]. In South Asia, terrorism threatened the sovereignty unity and territorial integrity of relevant countries in South Asia, and became an important excuse for external forces to violate the sovereignty of relevant countries and to interfere in the internal affairs of sovereign countries. Among them, there were threats from both external terrorist factors and internal separatist forces that tended to adopt terrorist means [59].

Most of the BRI countries were in developing countries. Although poverty did not directly lead to terrorist attacks, the interaction of economic, political, social, and security factors did increase the probability of terrorist attacks. The BRI had proposed “interconnection and interconnection,” which was the consensus reached by all BRI countries, to achieve policy, trade, financial flows, and people-to-people exchanges. Therefore, the BRI countries should strengthen cooperation with China and should strengthen international anti-terrorism cooperation, making great contributions to national security, economic development, and social stability of all countries.

### Effectiveness, limitations, and future directions

Our research closely linked BRI terrorist attack to the time and space. We identified the resolution of the incident by wavelet analysis and verified the feasibility of the honeycomb hexagon. The honeycomb hexagon was the best topological structure covering a two-dimensional plane, which reflected the spatial distribution trend of terrorist attacks in detail. Additionally, we used the GeoDetector to analyze the driving forces, which could reject the hypothesis of collinearity. Our research provided one reference for BRI national anti-terrorist operation. All BRI countries must work closely together to promote BRI. Only in this way can they achieve win-win results in all aspects.

Our study had some shortcomings. First, terrorist attacks are not simple crimes; they are complex and diverse. To analyze the spatial and temporal distribution of terrorist attacks, we should consider the target, the purpose, and the duration of terrorist attacks. Second, the selection of driving factors should not only consider human factors but also natural factors, such as terrain conditions and meteorological conditions. Third, the prediction of terrorist attacks and machine learning models will be used in future research.

## Conclusion

Using the GTD, we analyzed the spatiotemporal characteristics of terrorist attacks in BRI countries from 1998 to 2017 in the form of contours and cellular hexagons. We next used GeoDetector to analyze the driving forces. The conclusions are as follows:

1. From 1998 to 2017, the number of terrorist attacks in BRI countries showed an overall rising trend and presented a three-stage fluctuation growth trend during 1998–2004, 2004–2011, and 2011–2017. In terms of the number of events, we found that Monday was the peak period, and Friday was the trough period. The peak months were January, May, July, and November, and the trough months were June and September. In terms of incident rates, we found that the incidence of terrorist attacks was higher from Monday to Thursday, and the lowest was on Friday and Saturday. In addition, we also found that the distribution of the incidence of terrorist attacks during the first 11 years was scattered, ranging from 2% to 34%, and the range was very large. Meanwhile, the distribution over the next nine years was more even, ranging from 5% to 10%. Moreover, the incidence of terrorist attacks in April/May and July/August was relatively high. Finally, using wavelet analysis, we found that the 10-year-cycle characteristics of terrorist attacks in BRI countries from 1998 to 2017 were significant. Therefore, we divided the 20-year period into two stages, 1998–2007 and 2008–2017, for further analysis.
2. We counted the number of terrorist attacks in each honeycomb hexagon cell using ArcGIS and obtained the spatial distribution of the first and second 10-year terrorism events. Many terrorist attacks occurred in the Middle East and South Asia in both periods, with Iraq having the most attacks. Terrorist attacks during the latter 10 years had a wider scope and were larger in number compared with the previous period. China, Russia, Saudi Arabia, and northeastern Europe saw increased terrorist attacks during 2008–2017.
3. Most terrorist attacks were bombings/explosions; changes in the number of such attacks from 1998 to 2017 were similar to those of all incidents. Armed attacks ranked second, and assassinations, kidnappings, and facility/infrastructure attacks were the lowest and showed small differences. Spatially, Iraq/Israel, Afghanistan/Pakistan, and northeastern India were the core areas for the five types of attacks. The differences lay in the number of core areas and the sizes of the surrounding areas.
4. We used the factor detection function in GeoDetector to calculate the contribution of the 12 indicators of the Fragile States Index to the number of terrorist attacks during 2008–2017. Among the 12 indicators, the top three were security apparatus, human flight and brain drain, and external intervention. These provided a certain explanation for the occurrence of terrorist attacks in terms of political, economic, and social aspects. In short, if state organs cannot control the spread of terrorism, people’s well-being cannot be guaranteed, and factors such as strong interference from foreign forces will promote the development of terrorism.
